# Einsatz von Phagen bei Nutztieren, in Lebensmitteln und in der Umwelt im Rahmen eines One-Health-Ansatzes – Potenziale und Herausforderungen

**DOI:** 10.1007/s00103-025-04055-z

**Published:** 2025-05-20

**Authors:** Sophie Kittler, Jens A. Hammerl

**Affiliations:** 1 Institut für Lebensmittelqualität und -sicherheit, Stiftung Tierärztliche Hochschule Hannover, Hannover, Deutschland; 2 Abteilung Biologische Sicherheit, Bundesinstitut für Risikobewertung (BfR), Diedersdorfer Weg 1, 12277 Berlin, Deutschland

**Keywords:** Bakteriophagen, One Health, Bekämpfung, Zoonosen, Antimikrobielle Therapie, Antibiotikaresistenz, Bacteriophages, One Health, Treatment, Zoonoses, Antimicrobial therapy, Antimicrobial resistance

## Abstract

Im Sinne eines ganzheitlichen Schutzes der Gesundheit von Mensch, Tier und Umwelt (One Health) werden neben den konventionellen Ansätzen zunehmend Alternativen gesucht, um das Auftreten unerwünschter Bakterien kontrollieren zu können. Während sich klassische Antibiotika durch ihr breites Wirkungsspektrum auszeichnen, wird ihr Einsatz aufgrund zunehmender Resistenzentwicklungen kritisch diskutiert. Darüber hinaus hat der Einsatz von Antibiotika häufig gravierende Auswirkungen auf das umgebende Mikrobiom, dessen natürliche Zusammensetzung nachhaltig verändert werden kann. Die Notwendigkeit einer antibiotikafreien und zielgerichteten Bekämpfung bakterieller Infektionserreger stellt derzeit alle One-Health-Sektoren vor eine Herausforderung. Mit wachsendem Interesse werden z. T. lange vernachlässigte Bekämpfungsstrategien wiederentdeckt, die im Idealfall sektorübergreifend zur Sicherung der Gesundheit beitragen können.

Bakteriophagen (Phagen) kommen in allen Ökosystemen vor und stellen eine vielversprechende Ressource für die gezielte Biokontrolle von Bakterien dar. Als Viren, die nur bakterielle Zellen infizieren, interagieren sie spezifisch mit ihren Bakterienwirten, um deren Stoffwechsel für ihre Vermehrung zu nutzen. Dies geht mit der Zerstörung der Bakterienzelle einher. Der Nutzen dieser natürlichen Räuber-Beute-Beziehung für die Bekämpfung bakterieller Infektionserreger ist seit Langem bekannt und umfassend untersucht worden. Die vorliegende Übersichtsarbeit fasst ausgewählte Studien zusammen, die das Potenzial und die Anwendungsmöglichkeiten, aber auch die Herausforderungen des Phageneinsatzes verdeutlichen. Aufgrund ihrer sektorübergreifenden Relevanz wird der Einsatz von Phagen bei landwirtschaftlichen Nutztieren, in Lebensmitteln und in der Umwelt beispielhaft dargestellt.

## Abstract (en)

In order to ensure the holistic protection of human, animal and environmental health (commonly referred to as One Health), alternatives to the available antimicrobial approaches are required. Although antibiotic drugs have an outstanding broad spectrum of activity, their use is under discussion due to increasingly occurring resistances. Furthermore, the utilisation of antibiotics can result in significant consequences for the affected microbiome, often irreversibly altering its natural composition. The necessity for the implementation of alternative and targeted control measures for bacterial pathogens (e.g. zoonoses) represents a significant challenge for all sectors within the One Health framework. With growing interest, control strategies, some of which have been neglected for centuries, are under discussion for use as valuable tool for safeguarding health across all sectors.

Bacteriophages (phages) represent a naturally occurring biological resource with promising potential for use in targeted biocontrol of specific bacteria. As bacterial viruses, they interact specifically with their bacterial hosts, invading them to utilize the bacterial metabolism for their replication, finally destroying the infected bacterial cell. The benefits of this natural predator–prey relationship for the control of bacterial pathogens have long been recognized and extensively studied. This review summarises selected studies on the use of phages, illustrating the potential and application possibilities, but also the challenges of phage use. Due to their cross-sectoral relevance, the use of phages in farm animals, food and the environment is presented as an example.

## Hintergrund

Die Bewertung und das Management von gesundheitlichen Risiken wurden über einen langen Zeitraum hinweg auf einzelne Sektoren oder Bereiche fokussiert (beispielsweise auf Human- oder Tiermedizin oder den Umweltschutz). Gegenwärtig wächst das Bewusstsein für die enge Verbindung zwischen den verschiedenen Sektoren – die Idee einer zusammenhängenden Gesundheit manifestiert sich zunehmend in der wissenschaftlichen Gemeinschaft. Eine adäquate Einbindung entsprechender One-Health-Ansätze erfordert die Zusammenarbeit interdisziplinärer Gemeinschaften aus unterschiedlichen Wissenschaftssektoren, um erfolgreiche und ggf. sektorübergreifende Präventions- und Interventionsmaßnahmen zu erreichen [1]. Dies erfordert jedoch die Identifikation angemessener Ansatzpunkte in den einzelnen Sektoren.

Ein Beispiel hierfür ist der 4. gemeinsame Inter-Agency Report der Europäischen Behörde für Lebensmittelsicherheit, des Europäischen Zentrums für die Prävention und die Kontrolle von Krankheiten und der Europäischen Arzneimittelbehörde. Dieser ersetzt die bisherigen Einzelsektorberichte durch einen gemeinsamen Bericht der Sektoren, da die Übertragung von antimikrobiellen Resistenzen keine Sektorengrenzen kennt [2]. Auf Ebene der internationalen Organisationen (Food and Agriculture Organization – FAO, World Organization for Animal Health – OIE, United Nations Environment Programme – UNEP und World Health Organization – WHO) besteht seit Mai 2021 eine interdisziplinäre Zusammenarbeit durch die Einrichtung des One Health High-Level Expert Panel (OHHLEP), das den One-Health-Ansatz von der Theorie in die Praxis bringen will. Dies soll durch die Implementierung von verbesserter Kommunikation, Koordination und Kollaboration sowie Kapazitätsaufbau geschehen. Die Zusammenführung der einzelnen Sektoren zu einem integrierten Ganzen erfordert Entscheidungen hinsichtlich der Implementierung von Präventions- und Interventionsmaßnahmen, welche sich oft gleichzeitig auf mehrere Einzelbereiche auswirken. So wirken sich Entscheidungen im Bereich lebensmittelproduzierender Tiere unter Umständen direkt oder indirekt auf die menschliche Ernährung und die Gesundheit aus [1].

Im Zusammenhang mit der Anwendung von Antibiotika kommt es vermehrt zu multipler Resistenzentwicklung. Dieses Phänomen ist nicht nur auf den Veterinär- und Humanbereich beschränkt, sondern betrifft zunehmend auch die Umwelt. Vor diesem Hintergrund ergeben sich Kontroversen hinsichtlich einer gleichzeitigen Anwendung neuartiger antimikrobieller Substanzen in allen 3 One-Health-Sektoren. Bei der Anwendung von Antibiotika findet bereits nach kurzer Zeit eine Resistenzentwicklung statt, deren Auswirkungen zeitverzögert auch in allen anderen Sektoren nachweisbar sind [3]. Auch der komplette Verzicht auf Antibiotika führt nicht zwangsläufig zu einem Verlust von Resistenzen. Im Hinblick auf die Lokalisierung entsprechender Resistenzdeterminanten, die oftmals gemeinsam auf übertragbaren Plasmiden lokalisiert sind, ist der dauerhafte Verlust von Antibiotikaresistenzdeterminanten langfristig eher unwahrscheinlich und es kann im Zuge eines entsprechenden Selektionsdruckes jederzeit wieder zum Aufkommen einer dominanten Population resistenter Bakterien kommen [3].

Bakteriophagen (kurz: Phagen) stellen seit über 100 Jahren eine vielversprechende Alternative zur Bekämpfung von pathogenen und unerwünschten Bakterien dar. Es handelt sich um Viren, die ausschließlich Bakterien bzw. Archaeen infizieren und zerstören können [4]. In den vergangenen Jahrzehnten haben Phagen zunehmend an Bedeutung gewonnen, da die Resistenzraten unter vielen bakteriellen Erregern, insbesondere den sogenannten ESKAPE-Bakterien (*Enterococcus faecium, Staphylococcus aureus, Klebsiella pneumoniae, Acinetobacter baumannii, Pseudomonas aeruginosa* und *Enterobacter* spp.), gestiegen sind. In der Human- und Tiermedizin zeigt sich vermehrt, dass für die Behandlung bestimmter Infektionen keine wirksamen Antibiotika mehr zur Verfügung stehen [5]. Die Anwendung von Phagen könnte eine wirksame Alternative sein, hat sich jedoch bislang, auch in rechtlicher Hinsicht, nicht als Standard in Europa etablieren können. Der Arbeitsbericht Nr. 206 des Büros für Technikfolgen-Abschätzung beim Deutschen Bundestag (TAB) gibt einen umfassenden Überblick über den Stand der Entwicklung sowie die Einsatzmöglichkeiten von Bakteriophagen in Medizin, Landwirtschaft und Lebensmittelwirtschaft, inkl. einer Analyse der unterschiedlichen regulatorischen Rahmenbedingungen [6].

Das Ziel dieses Übersichtsbeitrags ist es, beispielhaft und an ausgewählten Studien das Potenzial, die Anwendungsmöglichkeiten und die Herausforderungen des Phageneinsatzes in Lebensmitteln und bei landwirtschaftlichen Nutztieren darzustellen und zu diskutieren.

## Vorteile und Nachteile von lytischen Bakteriophagen

Spezifische Phagen können mit vergleichsweise geringem Aufwand aus allen One-Health-Bereichen gewonnen werden, da sie überall dort vorkommen, wo ihre Wirtsbakterien existieren. Die hohe Spezifität, die Phagen üblicherweise aufweisen, kann als Vorteil, aber auch als Nachteil betrachtet werden. Der Vorteil besteht darin, dass die Phagen ausschließlich bestimmte Bakterien infizieren und zerstören können, wodurch eine breite Änderung der Zusammensetzung des Mikrobioms vermieden wird.

Die hohe Spezifität der Phagen birgt jedoch auch Nachteile. So kann es bei einer Veränderung des Erregers (z. B. Veränderung der Oberflächenproteinstruktur) zu einer Unwirksamkeit der bereitgestellten Phagenpräparate während der Anwendung kommen. Zudem müssen unter Umständen mehrere Phagen kombiniert oder für einzelne Zeitpunkte und Fälle spezifische Phagen ausgewählt werden, um gegen die Erreger wirksam zu sein. Dabei gibt es Unterschiede zwischen den Phagen: Einige Phagen werden als polyvalent eingestuft und decken ein breites Spektrum verschiedener Bakterientypen einer Spezies ab (bspw. *Staphylococcus, Pseudomonas*). Andere Erreger hingegen (z. B. *Klebsiella, Acinetobacter*) sind in ihren spezifischen Oberflächenstrukturen äußerst heterogen oder variabel, sodass eine Vielzahl verschiedener (monovalenter) Phagen verfügbar sein muss, um die Diversität der Zielerreger abdecken zu können [7].

## Potenzial einer Phagenanwendung bei landwirtschaftlichen Nutztieren

Die Effektivität von Phagen zur Erregerbekämpfung wurde in einer Vielzahl von Studien untersucht. Die umfangreichste Studiensammlung im tiermedizinischen Sektor existiert für Phagenanwendungen in der Geflügelmast. Während die Antibiotikaanwendungen in den anderen Tierhaltungssystemen (Rind, Schwein) durch umfangreiches Monitoring und Maßnahmen zur Verbesserung der Infektionsprophylaxe bereits deutlich reduziert werden konnten, sind die Reduktionen im Geflügelbereich trotz umfangreicher Bemühungen weniger deutlich. Die Anwendung von spezifischen Phagen könnte helfen, Infektionsgeschehen ohne den Einsatz von Antibiotika zu kontrollieren und so den im Grundgesetz verankerten Tierschutz sicherzustellen, ohne im umgebenden Mikrobiom eine Resistenzbildung und -förderung zu riskieren. Damit könnte ein wirksamer Beitrag zur Reduktion des Antibiotikaeinsatzes geleistet werden.

Ein häufiger Grund für das Verabreichen von Antibiotika in der Geflügelmast sind Infektionen mit aviärpathogenen *Escherichia coli* (APEC), die auch ein zoonotisches Potenzial aufweisen [8]. Diverse Studien konnten die Wirksamkeit von Phagen gegen APEC nachweisen. Untersuchungen zu kombinierter Applikation *per os* und durch Aerosolspray in Hähnchen zeigten eine signifikante Schutzwirkung der Phagen in Hinblick auf Mortalität, Morbidität, pathologisch-morphologische Veränderungen und/oder Bakterienkonzentration [9, 10]. In einer Studie konnte gezeigt werden, dass eine Injektion von Phagen in embryonierte Hühnereier die geschlüpften Küken vor einer Infektion mit APEC schützen konnte [11]. Nach der Behandlung wies eines von 16 untersuchten *E.-coli*-Isolaten Resistenzen gegen alle 4 verabreichten Phagen auf, ein anderes war weiterhin vollständig empfänglich, während die anderen Isolate nur gegen einen Teil der Phagen eine Resistenz aufwiesen. Bei 2 Isolaten mit 3‑facher bzw. vollständiger Resistenz gegen die verabreichten Phagen gingen die Resistenzen mit einem deutlichen Abfall der bakteriellen Virulenz einher, wie im Embryo-Letalitäts-Assay gezeigt werden konnte [11]. Diese Ergebnisse zeigen, dass Phagen als wirksame und nachhaltige Alternative zu Antibiotika in der Geflügelhaltung eingesetzt werden können.

Ein weiterer Bereich, in dem Phagen aufgrund ihrer Spezifität, Vermehrungsfähigkeit und der rechtlichen Möglichkeit zum präventiven Einsatz hohes Potenzial zur Antibiotikaminimierung bieten, ist die Bekämpfung von Mastitiden bei Milchkühen. Mastitiden verursachen bei milchgebenden Kühen starke Schmerzen und bei den Landwirten hohe ökonomische Verluste. Sie stellen zudem ein Infektionsrisiko für den Menschen dar. Ein aus 3 *E.-coli*-spezifischen Phagen bestehender Cocktail konnte nach Verabreichung ins Euter die Ausscheidung von Erregern mit der Milch, die Symptome und die Entzündungsmediatoren signifikant senken und erreichte Effekte, die mit einer Antibiotikatherapie vergleichbar waren [12]. Durch den verantwortungsvollen Einsatz von Bakteriophagen in diesem Bereich könnte ein Wirksamkeitsverlust von Antibiotika aufgrund von Resistenzbildung verhindert werden, um sie als Therapeutika für die Humanmedizin zu erhalten.

Im Bereich der Schweinezucht und -mast wurden bisherige Untersuchungen zum Einsatz von Bakteriophagen vornehmlich mit dem Ziel durchgeführt, das Auftreten von Methicillin-resistentem *Staphylococcus aureus* (MRSA) und dessen Übertragung auf den Menschen zu reduzieren. Eine Feldstudie setzte Phagen in einem Schweinezucht- und -mastbetrieb mit dem Ziel ein, die Haut- und Schleimhautkolonisation von Schweinen mit MRSA herabzusetzen. Trotz hoher Dosierung der Phagen konnte keine Reduktion der Besiedlung beobachtet werden. Ein zweiter Versuch dieser Studie beinhaltete, den Phagencocktail 3‑mal täglich zu vernebeln und zusätzlich über das Tränkwasser zu verabreichen. Wieder konnte jedoch keine Eradikation der MRSA aus dem Stall erreicht werden. Trotz einer sehr guten Wirkung des Phagencockails gegenüber MRSA-Isolaten von dem Betrieb *in vitro *und einer insgesamt hohen Dosis konnten die komplexen Infektionsketten der symptomlosen MRSA-Besiedlung in diesem Versuch nicht unterbrochen werden [13]. Auch eine weitere Studie, die mit dem Ziel der Eradikation von MRSA in gesunden Trägerschweinen durchgeführt wurde, konnte nach 6‑maliger Phagenapplikation und weiterhin bestehender Empfänglichkeit der Bakterien sowie Phagennachweis in Nase und auf der Haut keine Reduktion der MRSA-Besiedlung nachweisen. Die Ursache für die mangelnden Effekte in beiden Untersuchungen könnte eine geringe Bakterienkonzentration auf Haut und Schleimhaut gewesen sein, die eine effektive Vermehrung und das Zusammentreffen von Phagen und Bakterien erschwerte [14].

Zu klinischen Erkrankungen von Schweinen wurden erste Studien mit dem Ziel der Reduktion von *Bordetella bronchiseptica* durchgeführt, einem Erreger, der bei jungen Schweinen schwere respiratorische Erkrankungen hervorruft. Eine *In-vivo-*Studie demonstrierte nach der Applikation von Phagen antibakterielle und antiinflammatorische Effekte des verabreichten *Bordetella-bronchiseptica*-spezifischen Phagen [15].

Aufgrund der zu erwartenden Ernährungswende werden zudem weitere Nutztierhaltungssysteme wie Aquakulturen und Insektenhaltung immer relevanter. Auch hier bieten Phagen die Möglichkeit, die Tiere ohne den Einsatz von Antibiotika zu behandeln und die Bestände gesund zu erhalten, ohne Resistenzen gegenüber Antibiotika zu fördern. Signifikant reduzierte Mortalitätsraten konnten durch den Einsatz von Phagen gegen *Pseudomonas plecoglossicida *und *Aeromonas hydrophila* in Fischen sowie *Vibrio harveyi *in Shrimp-Larven erreicht werden [16–18]. Der Einsatz von Phagen gegen *Flavobacterium-psychrophilum-*Infektionen von Regenbogenforellen zeigte dagegen nur bei intraperitonealer Applikation eine signifikante Senkung der Mortalitätsraten [19]. Trotz des Potenzials ist bei der Verwendung von Phagen in Aquakulturen, wie bei der Nutzung von Antibiotika auch, eine Nutzen-Risiko-Analyse zum Austrag der Phagen in die Umwelt im Sinne eines One-Health-Ansatzes wichtig. Die Verbesserung der Tiergesundheit (z. B. durch Phagenapplikation oder angepasste hygienische Maßnahmen) kann zu einer Verringerung von multiresistenten Keimen führen, die durch den Kontakt zu Tieren oder den Konsum von tierischen Lebensmitteln auf den Menschen übertragen werden können [20].

## Kombination mit anderen Methoden im Lebensmittelbereich und Anwendung in der Umwelt

Eine weitere Erregergruppe, für die umfangreiche Untersuchungen zum Einsatz von Phagen durchgeführt wurden, sind Bakterien, die vom Tier auf den Menschen übertragen werden können (sogenannte Zoonosen, z. B. *Campylobacter, Salmonella, Listeria, Yersinia, Vibrio*), beispielsweise durch direkten Kontakt oder den Konsum von tierischen Produkten. Der Einsatz von Phagen wurde bereits umfassend für verschiedene Zielbakterien (hauptsächlich *Salmonella enterica, Listeria monocytogenes, Campylobacter *spp.) untersucht, wobei eine teilweise beeindruckende Senkung des Vorkommens der entsprechenden Zielbakterien durch den Einsatz von Phagen festgestellt wurde [2, 21, 22]. Die Gefahr einer Resistenzentwicklung gegenüber den eingesetzten Phagen besteht grundsätzlich immer, z. T. auch beim Einsatz eines Phagencocktails. Mit einer gezielten Kombination von verschiedenen Verfahren können jedoch die Vorteile unterschiedlicher Ansätze genutzt werden [23].

Vielversprechend können insbesondere Verfahren sein, die auf unterschiedlichen Wirkweisen beruhen. So könnte man z. B. Phagen anwenden und deren Keimzahlreduktionspotenzial über einen Temperaturanstieg zusätzlich fördern. Auch die kombinierte Anwendung von Phagen und nichtthermischen Verfahren, wie z. B. Hochdruck, Strahlung, Plasma, wäre möglich [24, 25, 30]. Durch das Zusammenwirken von Maßnahmen können Synergieeffekte erzielt werden, z. B. eine erhöhte Lyse durch Zellwanddestabilisierung. Detaillierte Studien zur Effizienz kombinierter Anwendungen fehlen oftmals oder sind nur mit einzelnen Stämmen durchgeführt worden. Hier wären weiterführende Forschungsarbeiten wünschenswert.

Des Weiteren sind Anwendungen von Phagen im Umweltbereich denkbar. Beispielsweise können sie zur Bekämpfung von Pflanzenpathogenen oder bedeutenden humanpathogenen Erregern in der Umwelt eingesetzt werden. Dazu zählen unter anderem *Vibrio cholerae* im Wasser, *Bacillus anthracis *auf bzw. in Böden sowie fäkale Kontaminanten (z. B. Shiga-Toxin-kodierende *Escherichia coli* (STEC), *Salmonella*) auf Lebensmitteln [25, 26].

## Verantwortungsvolle Nutzung von Phagen im One-Health-Kontext

Phagen lassen sich vielseitig einsetzen und sind aufgrund ihrer Diversität für unterschiedliche Anwendungen in großer Zahl verfügbar. Infolgedessen stellt sich die Frage, wie der Einsatz von Phagen im Sinne eines One-Health-Gedankens angemessen ist. Wie im Artikel von Willy und Bröcker zur Phagentherapie dargestellt, werden Phagen im humanmedizinischen Bereich zunehmend als „individueller Behandlungsversuch“ eingesetzt, auch wenn sie bisher nur in Ausnahmefällen angewandt werden.

Bei einem unkontrollierten Einsatz von Phagen im Human‑, Tier- oder Umweltbereich besteht die Gefahr, dass phagenresistente Bakterienstämme verbreitet werden, die zur Unwirksamkeit notwendiger Behandlungsmaßnahmen führen können. Einige Untersuchungen zur Resistenzentwicklung von Bakterien nach oraler Phagenbehandlung weisen zwar darauf hin, dass sich phagenresistente Bakterienpopulationen aufgrund von Fitnessverlusten nicht dauerhaft etablieren können, weitere Untersuchungen und ein Monitoring der bakteriellen Resistenzentwicklung sollten jedoch in Hinblick auf die Erfahrungen in der Antibiotikatherapie unbedingt bei jeder Behandlung durchgeführt werden. Dies vorausgesetzt, kann ein gezielter Phageneinsatz im Veterinärmedizin- oder Umweltbereich als potenzielle Interventionsmaßnahme in Betracht gezogen werden. Auch der rechtliche Rahmen für eine Zulassung in der Tiermedizin wurde geschaffen [27]. Phagen sind in diesem Zusammenhang vor allem dort von Interesse, wo sie gegen Erreger eingesetzt werden können, die beim Tier schwer therapierbare Erkrankungen hervorrufen oder die derzeit mit Antibiotikaklassen behandelt werden, die der Humanmedizin vorbehalten sind oder sein sollten.

## Nachhaltige Nutzungskonzepte für die Phagenanwendung

Wie bei jeder Räuber-Beute-Beziehung kann sich im Überlebenswettkampf von Phagen und Bakterien eine resistente Subpopulation bilden. Inwieweit diese langfristig in der Umwelt, im Tier oder im Menschen überlebensfähig sein wird, ist nur unzureichend untersucht. Hierzu gibt es unterschiedliche Erkenntnisse – etwa, dass resistente Bakterien nach kurzer Zeit ihre natürliche Empfindlichkeit gegenüber den Phagen zurückgewinnen. Andere Phagenmutanten zeigten genomische Veränderungen, die irreversibel waren. Unabhängig von der Dauer des Einflusses kann jede Veränderung der Zielbakterien (Erregeradaptation) einen Einfluss auf die Effizienz der Phagen haben [28]. Somit besteht ein kontinuierlicher Bedarf, neue Phagen zu isolieren und zu charakterisieren. Gerade im Bereich der Zulassung von Phagen für verschiedene Anwendungen wirft dies Probleme auf, da immer wieder Einzelfallentscheidungen auf Basis von Risikobewertungen getroffen werden müssen. Hier muss mit klaren Regularien ein Weg gefunden werden, der praxistauglich ist. Auch ein Einsatz von genetisch modifizierten Phagen sollte objektiv in Betracht gezogen werden, insbesondere wenn durch die genetische Veränderung z. B. der Wirtsbereich des effizienten Phagen auf einen spezifischen Erregertyp angepasst werden kann. Die genetische Anpassung von Phagen führt jedoch wieder zu regulativen Hürden, die gegen eine Anwendung von Phagen in bestimmten Bereichen sprechen (z. B. Lebensmittelsektor).

## Alternative phagenbasierte antimikrobielle Wirkstoffe

Neben dem Einsatz von Phagen sollte auch über alternative Bekämpfungsstrategien nachgedacht werden. Diese könnten phagenbasiert sein, wie z. B. Tailocine, phagenähnliche Partikel, die von Bakterien gegen konkurrierende Bakterien gebildet werden, oder phagenkodierte Enzyme (Depolymerasen, Lysine, Holine; [29]). Ein vielversprechender Ansatz ist der Einsatz von hydrolytischen Enzymen (Lysinen), welche die bakterielle Peptidoglykanschicht zerstören und letztlich zur Zelllyse führen können. Aufgrund ihrer effizienteren Wirkung gegen grampositive Zielbakterien (z. B. MRSA, *Listeria monocytogenes*) weist der Einsatz von Lysinen in diesem Bereich ein größeres Potenzial auf als bei gramnegativen Bakterien [30]. Die Limitationen von natürlichen Lysinen hinsichtlich ihrer Löslichkeit, lytischen Aktivität bzw. ihres engen Wirkungsspektrums können durch Anpassungen wie die Herstellung chimärer Varianten reduziert werden (für weiterführende Informationen siehe auch Beitrag von Idelevich und Becker in diesem Themenheft).

Eine weitere Möglichkeit ist die kombinierte Anwendung von Lysinen und antimikrobiellen Substanzen mit synergetischer Wirkung [29]. Die entsprechenden Möglichkeiten zur Nutzung etwaiger Enzyme bzw. chimärer Derivate werden von Behera et al. (2024) zusammenfassend erörtert [31]. Bei Mayorga-Ramos et al. geht es um ihren Einsatz insbesondere im Zusammenhang mit der Bekämpfung von Biofilmen bzw. einen präventiven Einsatz zur Vermeidung der Biofilmentwicklung in der Lebensmittelindustrie, in Milch‑/Molkereibetrieben oder auch in der Produktion medizinischer Erzeugnisse [32]. Ziel ist es, eine langfristig nachhaltige Entwicklung zu fördern und wirtschaftliche Verluste zu vermeiden.

## Fazit

Bakteriophagen sowie phagenassoziierte Enzyme können als gezielte Bekämpfungsstrategie gegen pathogene Bakterien zum Einsatz kommen. Der jeweilige Anwendungsbereich spielt für die Effizienz des Phageneinsatzes eine untergeordnete Rolle, sofern die erforderlichen Bedingungen für eine Phagenvermehrung oder Enzymwirkung bei den Zielbakterien gegeben sind. Die dargestellten Eigenschaften von Phagen liefern umfassende Argumente für einen gezielten Einsatz, wenn die Herausforderungen im Sinne eines One-Health-Ansatzes ausreichend adressiert werden. So ist beispielsweise eine kontinuierliche Anpassung der Phagenpräparate nötig, um den spezifischen Applikationen gerecht zu werden und mit dem stetigen Erregerwandel Schritt halten zu können. Zu den Herausforderungen zählt zudem die Entwicklung geeigneter Applikationsstrategien, welche die Effizienz der Phagen maßgeblich beeinflussen. Große Herausforderungen liegen insbesondere in den Regularien, die für die Zulassung bzw. die Nutzung der Phagen in der Praxis erforderlich sind. Insbesondere im Lebensmittelsektor sind weitere Anstrengungen erforderlich, um den Phageneinsatz als sinnvolle und sichere bakterielle Reduktionsmaßnahme zuzulassen. Ob Phagen tatsächlich das Gleichgewicht des vorliegenden (natürlichen) Mikrobioms im Sinne des One-Health-Ansatzes beeinflussen, ist bislang nicht ausreichend erforscht und bedarf weiterer Untersuchung.
